# Supplementary material for: ProteinShader: illustrative rendering of macromolecules
Source: BMC Struct Biol. 2009 Mar 30;9:19. doi: 10.1186/1472-6807-9-19 (PMC2672931; doi:10.1186/1472-6807-9-19)
Supplement: Additional file 1 — ProteinShader program without source code. This compressed file contains the complete ProteinShader program including associated libraries, but no source code. A README.txt file gives an overview of the ProteinShader distribution, and the index.html file in the help subdirectory has directions on getting started with the program as well as a set of tutorials. [file 1472-6807-9-19-S1.zip › ProteinShader-beta-0_9_4-binary/help/api/org/proteinshader/graphics/package-summary.html]

org.proteinshader.graphics (ProteinShader API)


|  |  |  |
| --- | --- | --- |
| **Overview** \| **Package** \| Class \| **Use** \| **Tree** \| **Deprecated** \| **Index** \| **Help** | |  |
| PREV PACKAGE   **NEXT PACKAGE** | **FRAMES**    **NO FRAMES**     **All Classes** |  |


---

## Package org.proteinshader.graphics

Holds the drawing classes: Ribbon, Tube, FrenetFrames, Sphere, and Cylinder.

**See:**
  
          **Description**

| **Class Summary** | |
| --- | --- |
| **Cylinder** | Knows how to draw a simple cylinder useful for representing the bonds between atoms. |
| **ExtrudedShape** | Contains common methods needed by classes that draw segments of a three-dimensional tube by sweeping a waist polygon along a spline. |
| **FrenetFrames** | Provides a visual representation of the LocalFrame objects of a Segment by drawing an x-axis, a y-axis, and z-axis. |
| **Ribbon** | Draws a segment of a three-dimensional ribbon by using a rectangular-shaped waist polygon. |
| **Shape** | Contains common methods needed by classes that draw geometric objects. |
| **Silhouette** | Uses jitter to repeatedly render a translucent black silhouette of a Drawable object for the purpose of antialiasing (smoothing jagged edges). |
| **Sphere** | Knows how to draw a sphere with texture coordinates, a surface normal, and a tangent vector for each vertex. |
| **Tube** | Draws a segment of a three-dimensional tube by using a waist polygon that approximates a circle as the number of vertices in the waist polygon becomes large. |

## Package org.proteinshader.graphics Description

Holds the drawing classes: Ribbon, Tube, FrenetFrames, Sphere, and Cylinder.
